# Fibroblast growth factor 23 is upregulated in the kidney in a chronic kidney disease rat model

**DOI:** 10.1371/journal.pone.0191706

**Published:** 2018-03-08

**Authors:** Hidekazu Sugiura, Ai Matsushita, Mayuko Futaya, Atsuko Teraoka, Ken-ichi Akiyama, Noriyoshi Usui, Nobuo Nagano, Kosaku Nitta, Ken Tsuchiya

**Affiliations:** 1 Fourth Department of Internal Medicine, Tokyo Women’s Medical University, Shinjuku, Tokyo, Japan; 2 Department of Nephrology, Division of Medicine, Saiseikai Kurihashi Hospital, Kuki, Saitama, Japan; 3 Division of Development of Mental Functions, Research Center for Child Mental Development, University of Fukui, Fukui, Japan; 4 Division of Developmental Higher Brain Functions, Department of Child Development, United Graduate School of Child Development, Osaka University, Osaka, Japan; 5 Kidney Disease and Dialysis Center, Hidaka Hospital, Hidaka-kai, Takasaki, Gunma, Japan; 6 Department of Medicine, Medical Center East, Tokyo Women’s Medical University, Arakawa, Tokyo, Japan; 7 Department of Blood Purification, Tokyo Women’s Medical University, Shinjuku, Tokyo, Japan; The University of Manchester, UNITED KINGDOM

## Abstract

The hormone fibroblast growth factor 23 (FGF23) is secreted from bone and is involved in phosphorus (P) metabolism. FGF23 mainly binds the FGF receptor, which interacts with αKlotho in the kidney or parathyroid and regulates Na-dependent phosphate co-transporter type IIa (NaPi-IIa) and type IIc (NaPi-IIc) expression, 1,25-dihydroxyvitamin D_3_ (1,25(OH)_2_D_3_) activity, and parathyroid hormone (PTH) secretion. In this study, we utilized hemi-nephrectomized rats fed a high-P diet (HP Nx), rats subjected to a partial nephrectomy (PN) and rats with doxorubicin-induced renal failure (DXR) as chronic kidney disease (CKD) animal models and analyzed the P metabolism and FGF23 expression in the kidneys in each CKD model. We cultured HK2 cells with a high level of P, 1,25(OH)_2_D_3_ or transforming growth factor-β1 (TGFβ1) to investigate the FGF23 expression mechanism. In both the HP Nx and PN rats, the blood FGF23 and PTH levels were increased. However, the 1,25(OH)_2_D_3_ level was increased in the HP Nx rats and decreased in the PN rats. In all three animal models, the mRNA expression of *αKlotho*, *NaPi-IIa* and *NaPi-IIc* was decreased, and the mRNA expression of *TGFβ1*, *collagen1a1*, *osteopontin* and *FGF23* was elevated in the kidney. FGF23 protein and mRNA were expressed at high levels in the extended tubule epithelium, which was an osteopontin-positive region in the HP and PN rats. *FGF23* and *osteopontin* mRNAs were expressed in HK2 cells incubated with TGFβ1; however, these levels were not altered in HK2 cells incubated with 1,25(OH)_2_D_3_ and high P levels in vitro. Altogether, FGF23 is expressed in the kidneys in CKD model rats. Following stimulation with TGFβ1, the injured renal tubular epithelial cells are strongly suspected to express both FGF23 and osteopontin. FGF23 produced in the kidney might contribute to P metabolism in subjects with CKD.

## Introduction

Fibroblast growth factor 23 (FGF23) is considered a causative gene in tumor-induced osteomalacia (TIO), including low-phosphorus (P) rickets/osteomalacia with P reabsorption failure in the renal tubules and autosomal dominant hypophosphatemic rickets/osteomalacia (ADHR) [1,2]. FGF23-transgenic mice have symptoms similar to those of hypophosphatemic rickets [3]. In addition, FGF23-deficient mice exhibit a failure to grow, bone lesions, a short lifespan, hyperphosphatemia, and high blood vitamin D levels, suggesting that FGF23 is a hormone regulator of P metabolism [4].

The phenotypes of FGF23-deficient mice and αKlotho-deficient mice are similar, and αKlotho is required for FGF23 to bind the fibroblast growth factor receptor (FGFR) [5]. In addition to FGFR, FGF can bind heparin and heparan sulfate (HS). Since HS is abundant in the extracellular matrix, secreted FGF does not diffuse and functions as a paracrine and autocrine factor [6]. In contrast, endocrine FGF isoforms, such as FGF19 (FGF15 in mouse), FGF21 and FGF23, have extremely low affinities to HS and diffuse into the blood. αKlotho has a very high affinity to FGF23 and can substitute for HS when FGF23 binds FGFR. Since αKlotho displays organ-specific expression, *e*.*g*., in the kidney and parathyroid, FGF23 functions in a tissue-specific manner [6].

αKlotho expression is decreased in subjects with renal failure, such as renal ischemia [7,8], fibrosis [9], and chronic kidney disease (CKD) [10–12]. In contrast, the blood FGF23 level is elevated beginning in the early stage of CKD and further increases as renal function deteriorates [13]. A high concentration of FGF23 is related to a worsening of the prognosis and deterioration of left ventricular hypertrophy in patients with CKD [14–16]. These effects are considered FGF23 functions that are independent of αKlotho. The calcineurin-nuclear factor of activated T cells signaling pathway is involved in FGF23-induced left ventricular hypertrophy in an animal model [15].

FGF23 is produced in the bone of patients with CKD [3]. The FGF23 levels increase with P loading, elevated 1,25-dihydroxyvitamin D_3_ (1,25(OH)_2_D_3_) levels, reduced αKlotho expression and reduced parathyroid hormone (PTH) levels [17–20]. In addition, a phosphate-binder treatment decreases blood FGF23 concentrations in rats with CKD [21]. In this study, we determined the site and mechanism of FGF23 production in kidney tissues using three rat models of CKD and renal proximal tubular epithelial cells incubated with TGFβ1, 1,25(OH)_2_D_3_ or a high level of P.

## Materials and methods

### Experimental animal models

Sprague-Dawley rats and Wistar rats were purchased from CLEA Japan, Inc., Tokyo, Japan. The animals were provided free access to standard food and water and received care in strict accordance with the recommendations in the *Guide for the Care and Use of Laboratory Animals* of the National Institutes of Health, *Guidelines for Proper Conduct of Animal Experiments* of the Science Council of Japan and *Tokyo Women’s Medical University Animal Experiment Regulations*. The protocol was approved by the Committee on the Ethics of Animal Experiments of Tokyo Women’s Medical University (Permit Number: AE16-66*)*. All animals were anesthetized with isoflurane (induction 4–5%, maintenance 2–3%) for animals (Intervet, Tokyo, Japan) prior to surgery, and all efforts were exerted to minimize suffering. The urine collection, water intake and food intake were managed using a metabolic cage. The rats were subcutaneously injected with 0.01 mg/kg subcutaneous buprenorphine (Otsuka Pharmaceutical Co., Ltd., Tokyo, Japan) for analgesia immediately before the operation. Then, we performed an additional subcutaneous injection of buprenorphine after 6 and 24 hours if the animals did not move, drink or eat. We referred to the Animal Research: Reporting of In Vivo Experiments (ARRIVE) guidelines. The animals were housed in metabolic cages for 96 hours, and the first 24 hours were used for acclimatization. Urine samples collected within the first 24 hours were discarded. Then, we collected and measured the urine samples, body weight, food consumption and amount of water drank by each rat every 24 hours. We used the average values obtained over three days. The animals were sacrificed under deep anesthesia with isoflurane.

#### Hemi-nephrectomized rats fed a high-P diet

Sprague-Dawley rats were used for this experiment. The food for the rats was purchased from ORIENTAL YEAST CO., LTD. (Tokyo, Japan). In this experiment, the high-phosphate (HP) diet contained 2.0% P and 0.6% calcium (Ca), whereas the non-high-phosphate (NP) diet contained 0.35% P and 0.6% Ca.

We performed a right-side nephrectomy (Nx) or sham operation on 7-week-old rats at week -2. After two weeks (9 weeks of age, week 0), the animals began the NP or HP diet. Seven weeks after the diet change (16 weeks of age, week 7), all animals were sacrificed and their blood, left kidney, calvaria and liver specimens were collected. The rats were divided into the following four groups: NP sham group (n = 8), NP Nx group (n = 7), HP sham group (n = 7) and HP Nx group (n = 9).

#### Partial nephrectomy rat model

Wistar rats were used in this experiment. Forty percent of the left kidney was surgically removed from 6-week-old rats in the mild partial nephrectomy group, and 60% of the left kidney was removed from the rats in the severe partial nephrectomy group. One week after the operation (7 weeks of age), nephrectomy of the right kidney or a sham operation was performed at SANKYO LABO SERVICE CORPORATION, INC. (Tokyo Japan). The rats were fed a standard diet, which contained 0.83% P and 1.07% Ca and was purchased from ORIENTAL YEAST CO., LTD. Nine weeks after the operation (16 weeks of age, week 9), all animals were sacrificed, and their blood, left kidney and liver specimens were collected. The rats were divided into the following three groups: sham group (n = 6), mild partial nephrectomy group (PN mild) (n = 6), and severe partial nephrectomy group (PN severe) (n = 6).

#### Rat model of doxorubicin-induced renal failure

In this experiment, we used frozen serum, frozen kidneys and kidney sections embedded in paraffin as described in our previous report [22]. The doxorubicin (DXR) used in this study was provided by Kyowa Hakko Kirin (Tokyo, Japan). The DXR-induced renal failure model was produced using a previously reported procedure [23]. Anesthetized rats were initially injected with 3 mg/kg of DXR via the tail vein, and another 2 mg/kg of DXR were injected after two weeks. The rats were divided into the vehicle group (n = 5) and DXR group (n = 5). Sixteen weeks after the initial DXR administration, all animals were sacrificed, and their blood and both right and left kidney specimens were collected.

### Measurement of biochemical parameters

The blood and urine samples were centrifuged at 1,300 ×g for 10 minutes. The serum was separated from the blood sample, and the urine supernatant was used. The creatinine (Cr), Ca and P levels were analyzed using an auto-analyzer at MONOLIS Co., Ltd. (Tokyo, Japan).

### Measurement of FGF23, 1,25(OH)_2_D_3_ and intact PTH levels

The serum concentrations of intact FGF23 were measured using an FGF23 ELISA kit (KAINOS Laboratories, Inc., Tokyo, Japan) according to the manufacturer’s recommended protocol. The serum 1,25(OH)_2_D_3_ levels were measured using the 1.25 (OH) 2D RIA Kit “TFB” (MONOLIS Co., Ltd.) according to the manufacturer’s instructions. The serum levels of intact PTH were measured using the Intact PTH ELISA kit (Immutopics, Inc., San Clemente, CA, USA) according to the manufacturer’s protocol.

### Cell culture

Human renal proximal tubular epithelial cells (HK2 cells) (#CRL-2190, American Type Culture Collection (ATCC), VA, USA) were grown to confluence in RPMI1640 medium (Thermo Fisher Scientific, Tokyo, Japan) on plastic dishes (Corning, Tokyo, Japan). The medium was supplemented with 10% fetal bovine serum (Thermo Fisher Scientific), 100 units/mL penicillin, and 100 μg/mL streptomycin (Thermo Fisher Scientific).

For the TGFβ1 stimulation, the HK2 cells were challenged with 0.5 ng/mL TGF-β1 (R&D Systems, Minneapolis, MN, USA) for 6, 24, 72 and 144 hours. For the 1,25(OH)_2_D_3_ stimulation, the HK2 cells were challenged with 0.4 μM 1,25(OH)_2_D_3_ (WAKO, Tokyo, Japan) for 6, 24, 72 and 144 hours. For the high-P stimulation, the HK2 cells were challenged with 2.5 mM β-glycerophosphate and 3 mM Na_2_HPO_4_/NaH_2_PO_4_ (P) medium (WAKO) for 1, 7 and 21 days [24].

### RNA isolation, reverse transcription, and real-time PCR

The total RNA was extracted from the kidneys, calvarias, livers and HK2 cells using TRIzol^®^ Reagent (Thermo Fisher Scientific) and an RNeasy Plus Mini Kit (QIAGEN, Tokyo, Japan). The cDNA templates were prepared using 2 μg of total RNA and a High-Capacity RNA-to-cDNA Kit (Thermo Fisher Scientific) and subjected to real-time PCR using a StepOnePlus^™^ Real-Time PCR System (Thermo Fisher Scientific). The primers used for the real-time PCR are listed in Table 1. The results were normalized to the cyclophilin level as previously described [9].

**Table 1:** Primers used for real-time PCR.

Gene	Forward primer	Reverse primer
Rat		
*FGF23*	AGGATGCTGGCTCCGTAGTG	GGCTGAAGTGATACGATCCAAA
*αKlotho*	CGTGAATGAGGCTCTGAAAGC	GAGCGGTCACTAAGCGAATACG
*NaPi-IIa*	CAGCATGACGTTTGTTGTCC	TCTGAAAGGAGCTGGAGAGC
*NaPi-IIc*	TGTCTGTCTGGTCCTCATCG	GTTAAGCCTGCTCCAACGAG
*CYP27B1*	CGAAGTTGCATAGGGAGACG	GGGTTTGACTGGAAGAGCAC
*TGFβ1*	CAACAATTCCTGGCGTTACC	CCCTGTATTCCGTCTCCTTG
*Col1a1*	TCGAGTATGGAAGCGAAGGT	TTGAGGTTGCCAGTCTGTTG
*Osteopontin*	CTCCAATGAAAGCCATGACC	AAACGTCTGCTTGTGTGCTG
*Osteocalcin*	CAAGTCCCACACAGCAACTC	AGGTCAGAGAGGCAGAATGC
*Cyclophilin*	ACGTGGTTTTCGGCAAAGT	CTTGGTGTTCTCCACCTTCC
Human		
*FGF23*	CTGGGACTGCTCTGGGTTAG	TGAGTCCTTGATGCCACTTC
*Osteopontin*	CAGAGTGCTGAAACCCACAG	TCAATCACATCGGAATGCTC
*E-cadherin*	TCTCACGCTGTGTCATCCA	ATTCGGGCTTGTTGTCATTC
*PAI1*	TTGCAGGATGGAACTACGG	GGCAGGCAGTACAAGAGTGA
*Cyclophilin*	TGCCATCGCCAAGGAGTAG	TGCACAGACGGTCACTCAAA

NaPi-IIa: Na-dependent phosphate co-transporter type IIa, NaPi-IIc: Na-dependent phosphate co-type IIc, CYP27B1: cytochrome P450 family 27 subfamily B member 1, TGFβ1: transforming growth factor-β1, Col1a1: collagen1a1, PAI1; plasminogen activator inhibitor-1

### Antibodies

The following antibodies were used as primary antibodies for the Western blotting: monoclonal anti-mouse FGF-23 antibody (#MAB26291, R&D Systems) [25] and monoclonal mouse glyceraldehyde 3-phosphate dehydrogenase (GAPDH) antibody (6C5) (#NB600-502, Novus Biologicals, Littleton, CO, USA). The primary antibodies used for the immunohistochemical staining and immunofluorescence staining included an anti-FGF-23 antibody (#MBS854462, MyBioSource, CA, USA) [26], a monoclonal anti-actin α-smooth muscle antibody produced in mouse (#A2547, Sigma-Aldrich, Tokyo, Japan), and a monoclonal mouse osteopontin antibody (#LB-4225LSL, Cosmo Bio Co, Tokyo, Japan).

### Western blot analysis

The frozen kidney samples were homogenized in lysis buffer (20 mM Na-HEPES [pH 7.5], 100 mM NaCl, 1% Triton X-100, 15 mM NaF, 1 mM Na_3_VO_4_, 10 mM Na_4_P_2_O_7_, 1 mM EDTA, Protease Inhibitor Cocktail ([Roche, Tokyo, Japan], and Phosphatase Inhibitor Cocktail 2 [Sigma-Aldrich]) on ice and centrifuged at 5,591×g for 10 minutes to remove debris. The protein concentrations in the supernatants were determined using 5 μg/μL. Ten micrograms of protein from each specimen were suspended in loading buffer, separated on a 4–15% Criterion^™^ TGX^™^ Precast Gel (Bio-Rad, Tokyo, Japan), and electrophoretically transferred to a nitrocellulose membrane. The membrane was blocked with 5% skim milk in TBS and incubated with a 500-fold dilution of the primary antibodies overnight at 4°C. After three 5-minute washes with washing buffer (0.1% Tween 20 in TBS), the membranes were incubated with the secondary antibodies for 1 hour at room temperature. After three 10-minute washes with washing buffer, the membrane was incubated with Enhanced Chemiluminescence (ECL) Western Blotting Detection Reagents (GE Healthcare, Tokyo, Japan) and exposed using LuminoGraph (ATTO, Tokyo, Japan) and ImageSaver6 (ATTO). GAPDH was used as an internal control.

### Histological examinations

Hematoxylin-eosin (HE) staining, Masson’s trichrome (MT) staining (to evaluate fibrosis) and Von Kossa (VK) staining (to evaluate calcification) were performed using paraffin-embedded kidney sections.

### Immunohistochemistry

The kidneys were fixed with 4% paraformaldehyde in PBS for 16 hours at 4°C and then embedded in paraffin. The paraffin-embedded sections (3-μm-thick) were deparaffinized, hydrated and then incubated with 0.3% H_2_O_2_ in methanol for 30 minutes. A VECTASTAIN ABC Standard Kit (Vector Laboratories, Tokyo, Japan) was used for the immunohistochemical staining. The sections were blocked with normal blocking serum for 30 minutes and then incubated with a 500-fold dilution of the primary antibodies for 16 hours. After the incubation with the appropriate biotinylated goat anti-rabbit IgG secondary antibody for 60 minutes, the sections were stained with Vectastain ABC Reagent for 30 minutes, followed by the peroxidase substrate solution using the DAB substrate kit (Vector Laboratories).

### Immunofluorescence staining

The kidneys were fixed with 4% paraformaldehyde in PBS for 16 hours at 4°C, sequentially immersed in 10%, 15% and 20% sucrose in PBS for 12 hours at 4°C, embedded in optimum cutting temperature (OCT) compound and immediately frozen in liquid nitrogen.

The immunofluorescence staining was performed using a primary antibody and a secondary antibody (Alexa Fluor^®^ 488-conjugated goat anti-rabbit IgG (H+L) secondary antibody or Alexa Fluor^®^ 568-conjugated goat anti-mouse IgG (H+L) secondary antibody, [Thermo Fisher Scientific]). The sections were blocked for 1 hour and incubated with a 500-fold dilution of the primary antibody for 16 hours at 4°C. After the incubation with the secondary antibody for 1 hour at room temperature in the dark, the sections were incubated with DAPI (4’,6-diamidino-2-phenylindole, dihydrochloride) (Thermo Fisher Scientific) and mounted using VECTASHIELD Mounting Medium (Vector Laboratories). The images of the sections were captured using Axio Observer Z1 and Axio Vision (ZEISS, Tokyo, Japan).

### In situ hybridization

In situ hybridization was performed as previously described [26,27]. The kidneys were fixed with 4% paraformaldehyde in PBS for 16 hours at 4°C and then embedded in paraffin. RNAscope^®^ 2.5 HD Reagent kit-RED (#322350, Advanced Cell Diagnostics, Hayward, CA, USA) was used for the in situ hybridization. The paraffin-embedded sections (5-μm-thick) were treated according to the manufacturer’s instructions.

*FGF23* was detected using a rat-specific probe against bases 9–652 of its mRNA (RNAscope^®^ Probe- Rn-Fgf23, #484501, accession no. NM_130754.1). A probe against the housekeeping gene peptidylprolyl isomerase B (RNAscope^®^ Positive Control Probe- Rn-Ppib, #313921, accession no. NM_022536.2) and a probe targeting the bacterial gene *dapB* (RNAscope^®^ Negative Control Probe- DapB, #310043, accession no. EF191515) were used as positive and negative controls, respectively, to verify the mRNA quality. RNAscope^®^ Control Slide-Mouse 3T3 Cell Pellet (Cat. No. 310023) was used to verify the assay procedures.

### Statistical analysis

The data are presented as the means ± standard errors of the means (SEM). One-way ANOVA, two-way ANOVA, Kruskal-Wallis tests, unpaired t-tests with Bonferroni correction and Mann-Whitney U-tests with Bonferroni correction were performed using Excel (Microsoft WA, USA) and JMP for Windows (SAS Institute, NC, USA). P-values <0.05 represent statistically significant differences.

## Results

### Hemi-nephrectomized rats fed a high-P diet

#### Renal function and mineral metabolism

**Fig 1:**
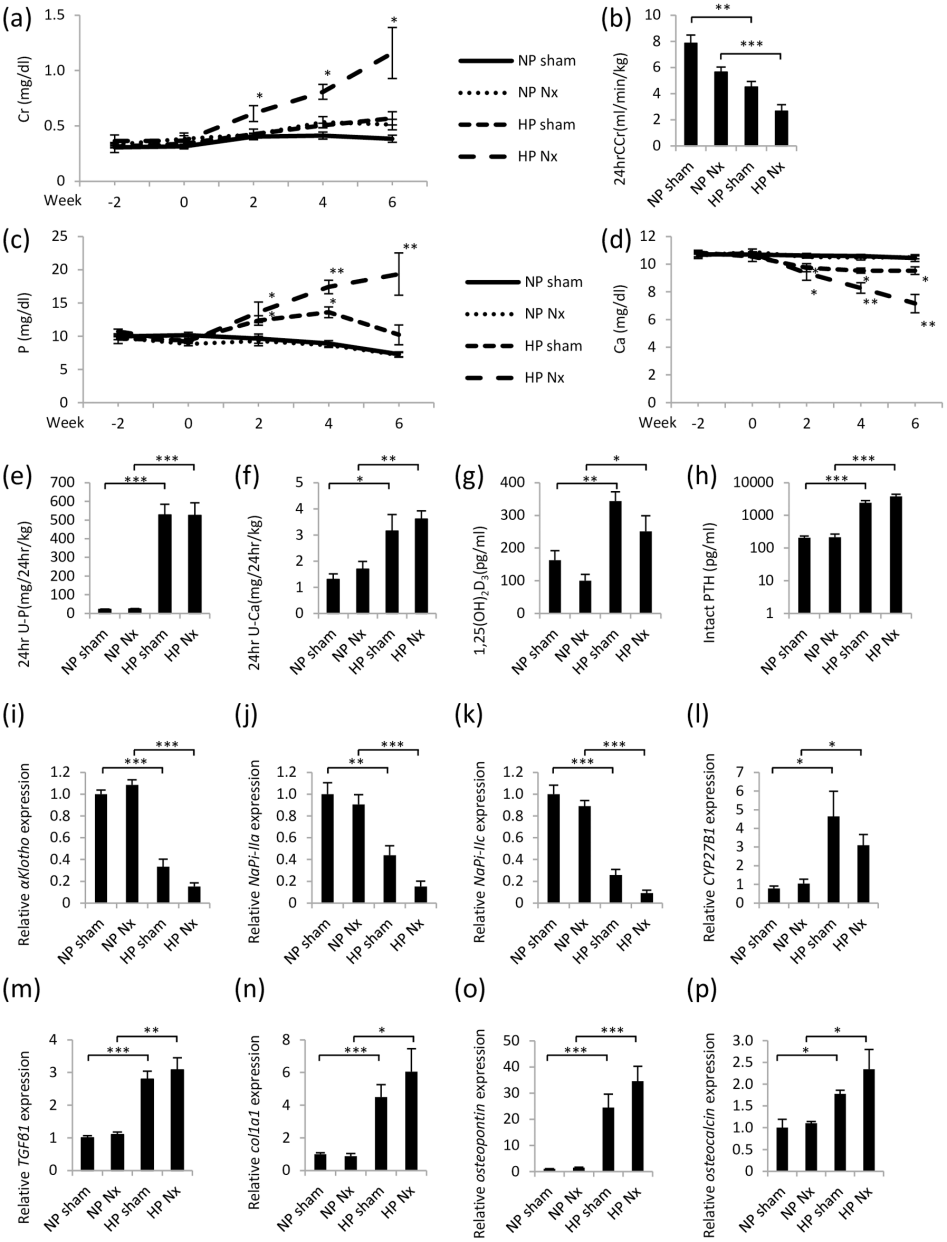
Hemi-nephrectomized rats fed a high-P diet. (a) Serum Cr concentration. (b) The 24-hr CCr was calculated using the following formula: (urine Cr concentration×24-hour urine volume)/(serum Cr concentration×1440×body weight). (c) Serum P concentration. (d) Serum Ca concentration. (e) The 24-hr U-P was calculated using the following formula: (urine P concentration x 24-hour urine volume)/body weight. (f) The 24-hr U-Ca was calculated using the following formula: (urine Ca concentration×24-hour urine volume)/body weight. (g) Serum 1,25(OH)_2_D_3_ concentration. (h) Serum intact PTH concentration. (i) *αKlotho* mRNA expression in the kidney. (j) *NaPi-IIa* mRNA expression in the kidney. (k) *NaPi-IIc* mRNA expression in the kidney. (l) *CYP27B1* mRNA expression in the kidney. (m) *TGFβ1* mRNA expression in the kidney. (n) *Col1a1* mRNA expression in the kidney. (o) *Osteopontin* mRNA expression in the kidney. (p) *Osteocalcin* mRNA expression in the kidney. Each value represents the mean ± SEM. *P<0.05, **P<0.01, ***P<0.001, NP sham group vs HP sham group or NP Nx group vs HP Nx group; NP sham group (n = 8), NP Nx group (n = 7), HP sham group (n = 7) and HP Nx group (n = 9). (i-p) NP sham group was used as a normalization control.

Regarding renal function, the serum creatinine (Cr) levels did not significantly differ between the HP sham group and NP sham group. The 24-hour Cr clearance (24-hr CCr) at week 6 was significantly lower in the HP sham group than that in the NP sham group. The serum Cr level was significantly higher in the HP Nx group than that in the NP Nx group. The 24-hr CCr was also significantly lower in the HP Nx group than that in the NP Nx group (Fig 1a and 1b).

The serum P levels detected in the HP Nx group were higher than those detected in the NP Nx group at all time points throughout the experiment. The serum P level increased at weeks 2 and 4 after the animals in the HP sham group started the high-P diet and then gradual decreased. At week 6, no significant differences were observed compared with the animals fed the NP diet. In contrast, the 24-hour urine P excretion (24-hr U-P) was higher in the HP groups than that in the NP groups. However, a significant difference was not observed between the HP sham group and HP Nx group. The serum Ca level was lower in the HP groups than that in the NP groups at all time points throughout the experiment. The 24-hour urine Ca excretion (24-hr U-Ca) was higher in the HP groups (Fig 1c–1f). The serum 1,25(OH)_2_D_3_ levels detected in the HP groups were higher than those detected in the NP groups. The serum level of intact PTH was higher in the HP groups than that in the NP groups (Fig 1g and 1h). The *NaPi-IIa* and *NaPi-IIc* mRNAs were measured because FGF23-αKlotho signaling suppresses NaPi-IIa and NaPi-IIc expression [6]. Compared with the NP groups, the *αKlotho*, *NaPi-IIa* and *NaPi-IIc* mRNAs were expressed at lower levels in the kidneys in the HP groups. In addition, the mRNA expression of *cytochrome P450 family 27 subfamily B member 1 (CYP27B1)*, which encodes 25-hydroxyvitamin D3 1-alpha-hydroxylase (1α-hydroxylase), showed changes that were similar to the changes in the 1,25(OH)_2_D_3_ levels (Fig 1i–1l). Transforming growth factor-β1 (TGFβ1), collagen1a1 (Col1a1), osteopontin and osteocalcin are markers of fibrosis and calcification. The *TGFβ1*, *Col1a1*, *osteopontin* and *osteocalcin* mRNAs expressed in the kidneys in the HP groups were higher than those expressed in the kidneys in the NP groups (Fig 1m–1p).

#### FGF23 expression

**Fig 2:**
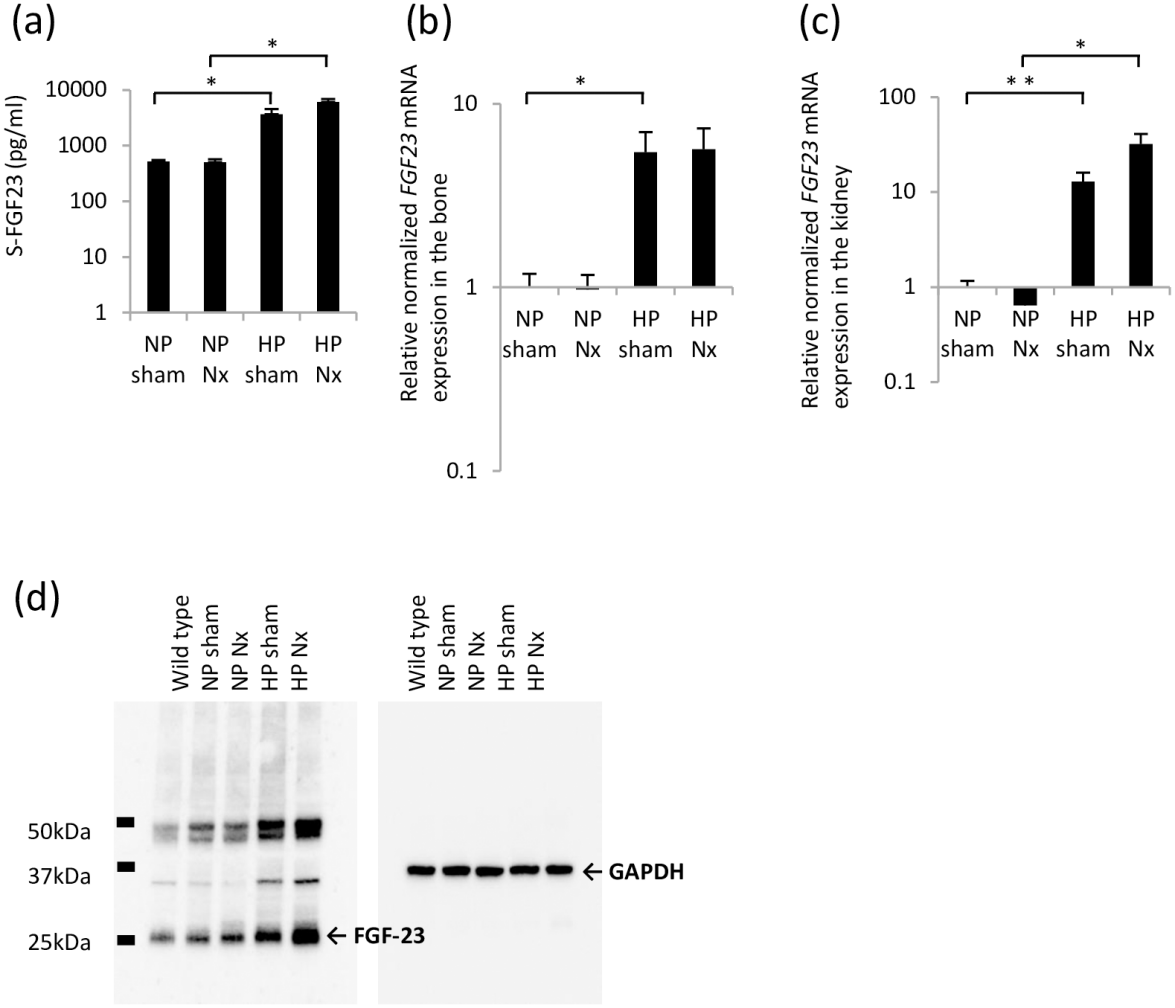
FGF23 expression in hemi-nephrectomized rats fed a high-P diet. (a) Serum FGF23 concentration. (b) *FGF23* mRNA expression in the bone. NP sham group was used as a normalization control. (c) *FGF23* mRNA expression in the kidney. NP sham group was used as a normalization control. (d) Western blot of FGF23 in the kidney. GAPDH was used as an internal control. Each value shown represents the mean ± SEM; *P<0.05, **P<0.01; NP sham group (n = 8), NP Nx group (n = 7), HP sham group (n = 7) and HP Nx group (n = 9).

The serum FGF23 levels detected in the HP groups were higher than those detected in the NP groups. The mRNA expression of *FGF23* was measured in the calvaria. *FGF23* mRNA in the bone specimens from the HP groups was expressed at higher levels than that in the NP groups (Fig 2a and 2b). The mRNA expression of *FGF23* was also measured in the kidney. Although *FGF23* mRNA was barely detectable in the kidneys from the NP groups, it was detectable in the kidneys from the HP groups. The difference between the two groups was statistically significant. We did not observe any significant differences between the HP sham group and the HP Nx group (Fig 2c). The expression of *FGF23* mRNA was not detected in the liver specimens from each group by real-time PCR (S3a Fig).

Therefore, the FGF23 protein levels in the kidney were measured by performing Western blotting. The levels of FGF23 protein were elevated in the kidneys in the HP sham group and HP Nx group (Fig 2d).

#### Renal histology

**Fig 3:**
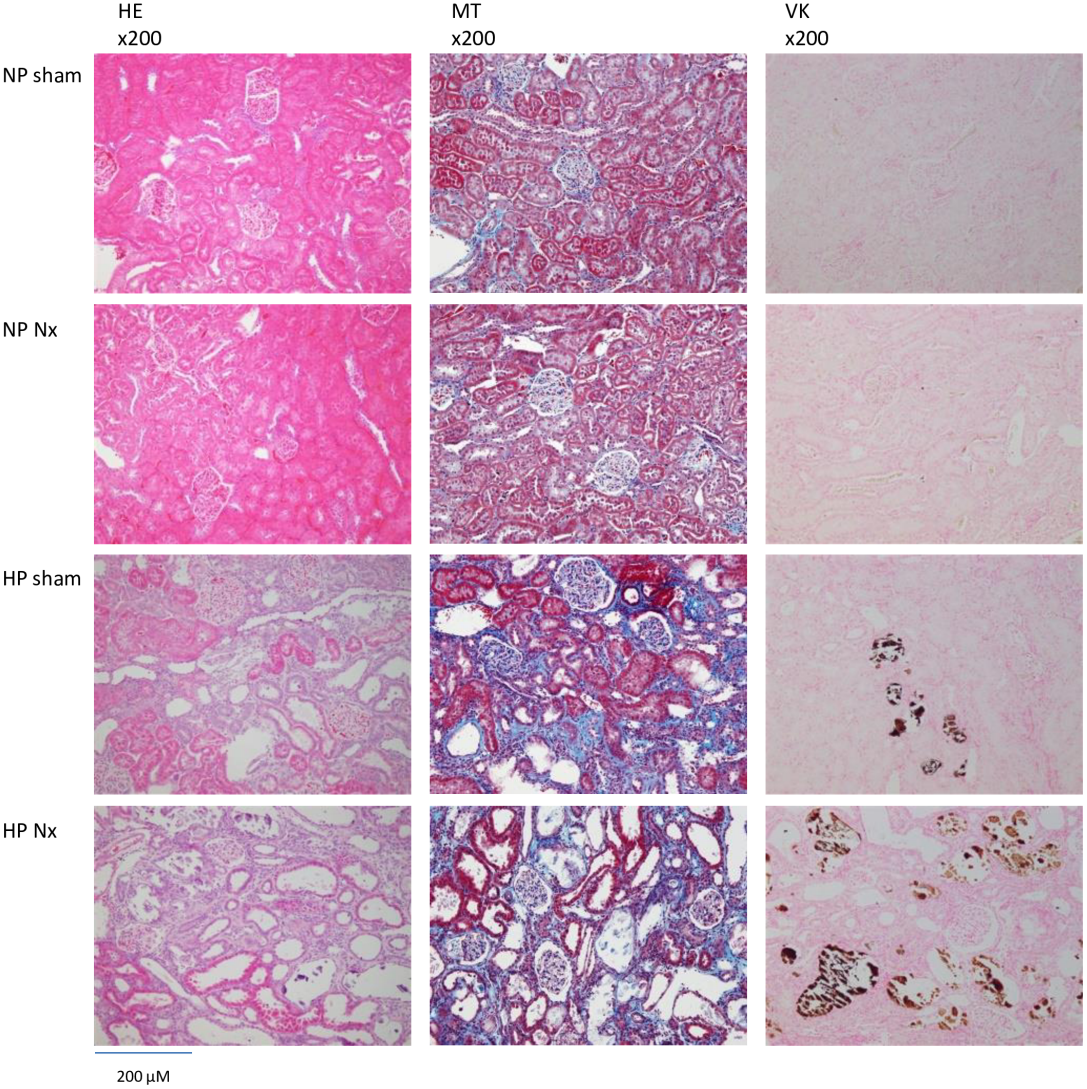
Histology on the kidney in hemi-nephrectomized rats fed a high-P diet. HE: hematoxylin-eosin staining. MT: Masson’s trichrome staining as an evaluation of fibrosis. VK: Von Kossa staining as an evaluation of calcification. ×200: high magnification.

**Fig 4:**
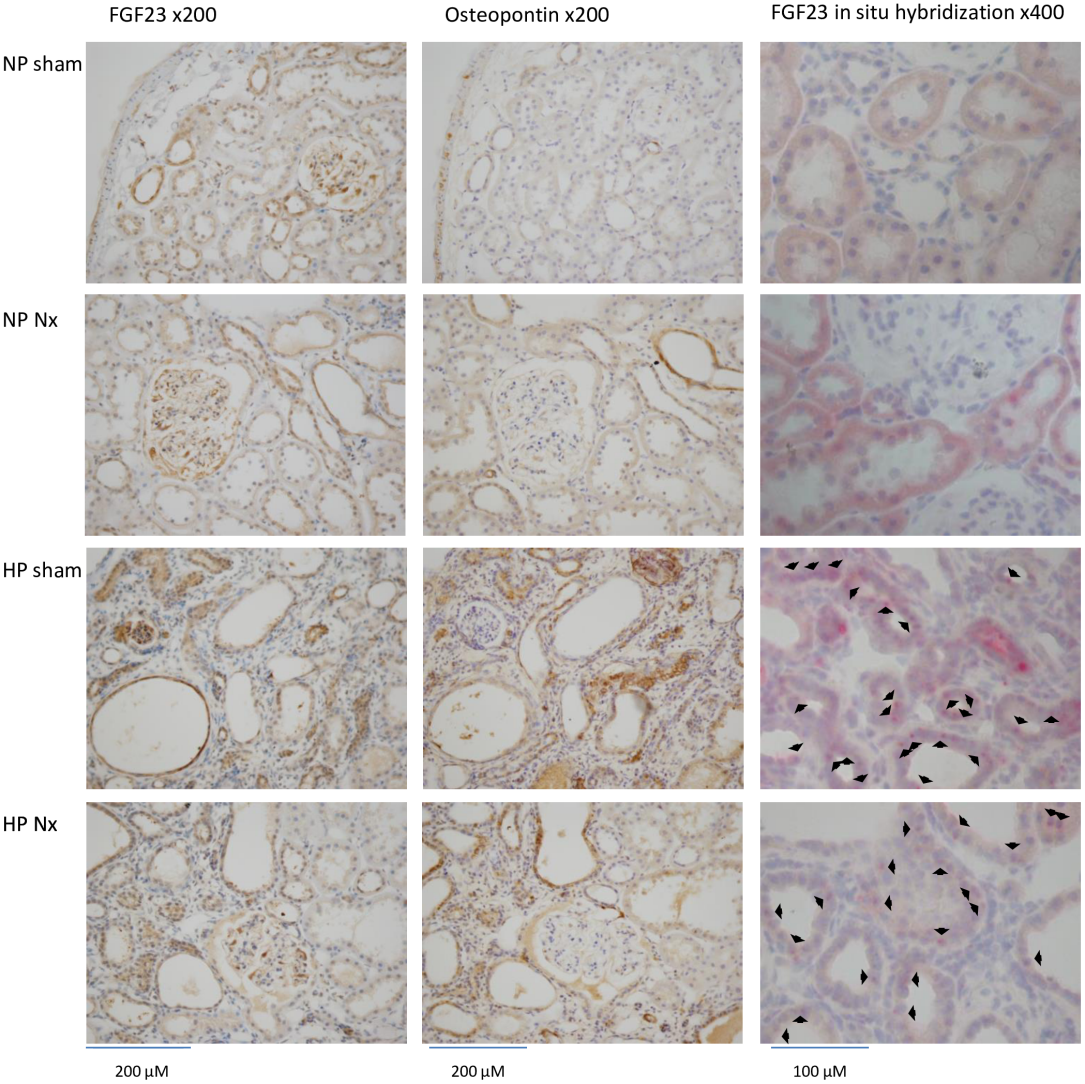
Immunohistochemistry and in situ hybridization in the kidney of hemi-nephrectomized rats fed a high-P diet. FGF23: FGF23 immunohistochemistry (brown). Osteopontin: osteopontin immunohistochemistry (brown). ×200: high magnification. *FGF23* in situ hybridization (red spots. arrow: positive cells). ×400: high magnification.

**Fig 5:**
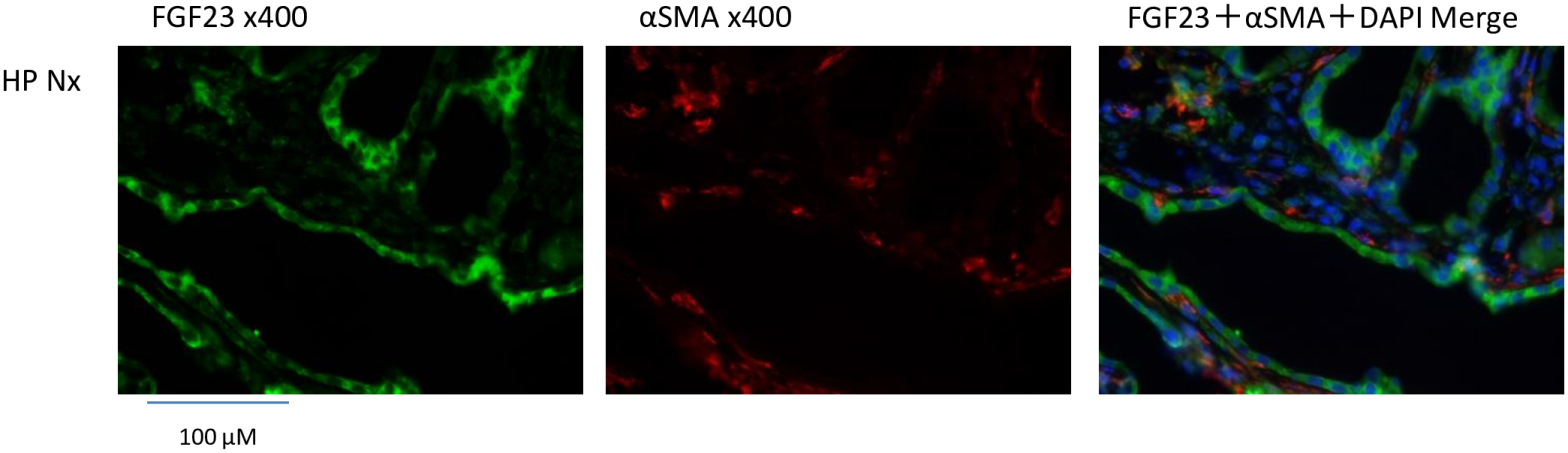
Immunofluorescence in the kidney of hemi-nephrectomized rats fed a high-P diet. FGF23 immunofluorescence (green). αSMA immunofluorescence (red). Merge: FGF23 (green), αSMA (red), and DAPI (blue). ×400: high magnification.

In the HP groups, we observed interstitial fibrosis in the outer medulla, calcification of the tubule lumen, expansion of the renal tubule lumen and a decreased number of tubule cells (Fig 3, HE, MT and VK). The immunohistochemical staining of FGF23 was detected in the expanded tubular cells in the HP groups. In the NP sham group, we also detected weak positive signals in several glomerular and tubular cells. The area of FGF23 staining was greater in the HP groups (Fig 4, FGF23). Osteopontin is a biomarker of bone matrix. Osteopontin was detected in the expanded tubular cells expressing FGF23 in serial sections of the kidney (Fig 4, osteopontin). αSMA is a biomarker of interstitial fibrosis, tubule cells undergoing the epithelial-mesenchymal transition (EMT) and vascular smooth muscle cells. We performed double immunofluorescence staining of αSMA and FGF23. In the HP groups, the FGF23 and αSMA signals were not merged in the expanded tubules (Fig 5, FGF23, αSMA and merge). According to the *FGF23* in situ hybridization, *FGF23* mRNA was detected in the expanded tubular cells in the HP groups. We did not detect positive signals in the NP sham group (Fig 4, *FGF23* in situ hybridization).

### HK2 cells treated with TGFβ1, 1,25(OH)_2_D_3_ or a high level of P

In the HP diet-fed rats, the renal FGF23 expression was increased under special circumstances, such as high P loading, high serum 1,25(OH)_2_D_3_ levels, fibrosis and calcification of the renal tubules. P loading and elevated 1,25(OH)_2_D_3_ levels have been reported to increase the FGF23 levels. Subsequently, we cultured HK2 cells with high levels of P, 1,25(OH)_2_D_3_ or TGFβ1 in vitro to investigate the mechanism underlying FGF23 expression in the renal tubules.

**Fig 6:**
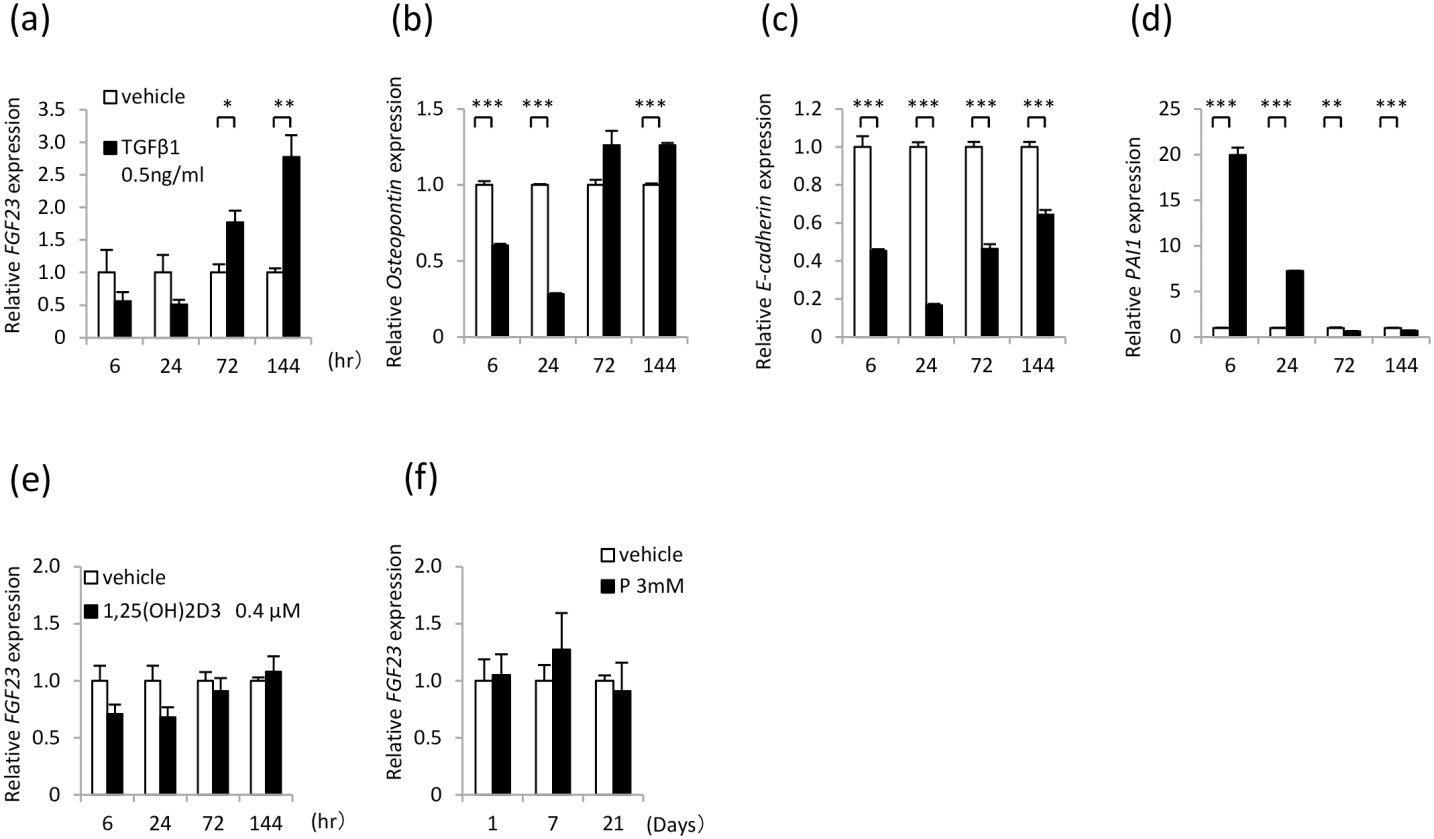
FGF23 mRNA expression in HK2 cells. (a) *FGF23* mRNA expression in HK2 cells stimulated with TGFβ1. (b) *Osteopontin* mRNA expression in HK2 cells stimulated with TGFβ1. (c) *E-cadherin* mRNA expression in HK2 cells stimulated with TGFβ1. (d) *PAI1* mRNA expression in HK2 cells stimulated with TGFβ1. (m) *FGF23* mRNA expression in HK2 cells stimulated with 1,25(OH)_2_D_3_. (l) *FGF23* mRNA expression in HK2 cells stimulated with high-P levels. Each value represents the mean ± SEM; *P<0.05, **P<0.01, ***P<0.001, compared with the vehicle (each group: n = 3). Vehicle group was used as a normalization control.

A TGFβ1 stimulation for 6 and 24 hours (short-term stimulation) tended to reduce the *FGF23* mRNA expression, but a TGFβ1 stimulation for 72 and 144 hours (long-term stimulation) significantly induced *FGF23* mRNA expression in the HK2 cells (Fig 6a). The *osteopontin* mRNA expression was also decreased in the cells subjected to the short-term TGFβ1 treatment but increased in the cells subjected to the long-term TGFβ1 treatment (Fig 6b). The *E-cadherin* mRNA expression was decreased by TGFβ1 (Fig 6c). The *PAI1* mRNA expression was increased in response to the short-term TGFβ1 stimulation but decreased in response to the long-term TGFβ1 stimulation (Fig 6d).

For the 1,25(OH)_2_D_3_ stimulation, the HK2 cells were challenged with 0.4 μM 1,25(OH)_2_D_3_ for 6, 24, 72 and 144 hours. For the high P stimulation, the HK2 cells were challenged with 3 mM Na_2_HPO_4_/NaH_2_PO_4_ (P) and 2.5 mM β-glycerophosphate medium for 1, 7 and 21 days. The 1,25(OH)_2_D_3_ and P stimulations did not change the *FGF23* mRNA expression levels (Fig 6e and 6f).

### Partial nephrectomy rat model

#### Renal function and mineral metabolism

Based on the results obtained in the in vitro HK2 cell-based model, TGFβ1 might induce FGF23 expression in animal models of CKD. Therefore, we used a partial nephrectomy rat model, which usually displays high TGFβ1 levels, normal serum P and 1,25(OH)_2_D_3_ levels, and a lack of calcification during the early and middle stages of CKD, and examined the FGF23 expression in the kidney.

**Fig 7:**
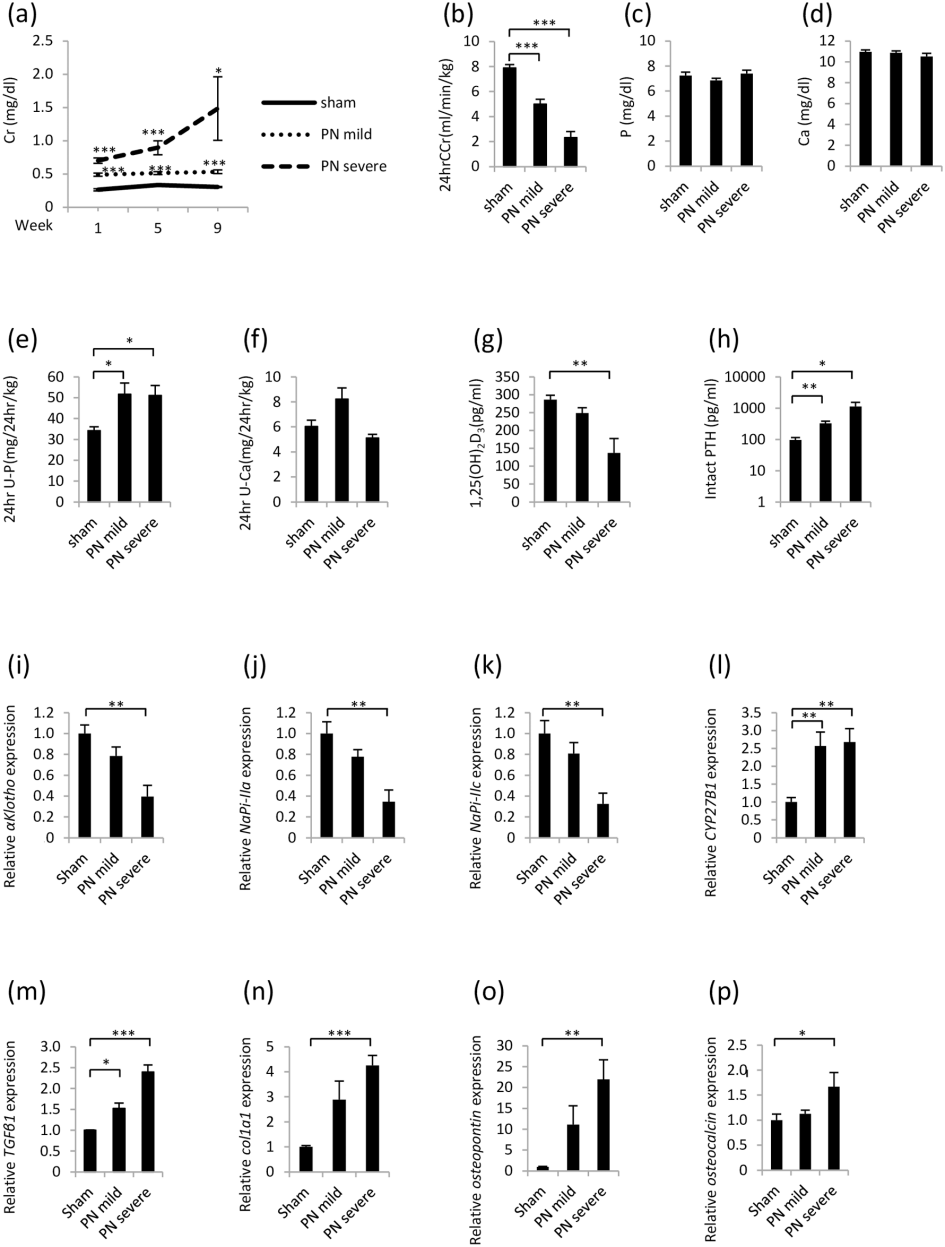
Partial nephrectomy rat model. (a) Change in serum Cr concentration. (b) The 24-hr CCr. (c) Serum P concentration. (d) Serum Ca concentration. (e) The 24-hr U-P. (f) The 24-hr U-Ca. (g) Serum 1,25(OH)_2_D_3_ concentration. (h) Serum intact PTH concentration. (i) *αKlotho* mRNA expression in the kidney. (j) *NaPi-IIa* mRNA expression in the kidney. (k) *NaPi-IIc* mRNA expression in the kidney. (l) *CYP27B1* mRNA expression in the kidney. (m) *TGFβ1* mRNA expression in the kidney. (n) *Col1a1* mRNA expression in the kidney. (o) *Osteopontin* mRNA expression in the kidney. (p) *Osteocalcin* mRNA expression in the kidney. Each value represents the mean ± SEM; *P<0.05, **P<0.01, ***P<0.001, sham group vs PN mild group or sham group vs PN severe group. Sham group (n = 6), PN mild group (n = 6), PN severe group (n = 6). (i-p) Sham group was used as a normalization control.

Regarding renal function, the serum Cr level was significantly higher in the PN mild and severe groups than that in the sham group. At week 9, the 24-hr CCr was also significantly lower in the PN mild and severe groups than that in the sham group (Fig 7a and 4b).

Although the serum P levels in the PN mild and severe groups did not differ from those in the sham group, the 24-hr U-P was higher in the PN mild and severe groups than that in the sham group at week 9. The serum Ca levels in the PN mild and severe groups did not differ from those in the sham group. At week 9, the 24-hr U-Ca in the PN mild and severe groups did not significantly differ from that in the sham group (Fig 7c–7f).

Although the serum 1,25(OH)_2_D_3_ level was lower in the PN mild group than that in the sham group at week 9, the difference was not significant. However, the serum 1,25(OH)_2_D_3_ level was significantly lower in the PN severe group than that in the sham group. The serum level of intact PTH was significantly higher in the PN mild and severe groups than that in the sham group (Fig 7g and 7h).

The *αKlotho*, *NaPi-IIa* and *NaPi-IIc* mRNAs were expressed at significantly lower levels in the kidneys of the PN severe group than those in the kidneys of the sham group. In addition, the expression of *CYP27B1* mRNA was significantly higher in the PN mild and severe groups than that in the sham group. The *CYP27B1* mRNA expression level did not correlate with the serum 1,25(OH)_2_D_3_ level (Fig 7i–7l). The levels of *TGFβ1*, *Col1a1*, *osteopontin* and *osteocalcin* mRNAs found in the kidneys of the PN severe group were higher than those found in the kidneys of the sham group (Fig 7m–7p).

#### FGF23 expression in the kidney

**Fig 8:**
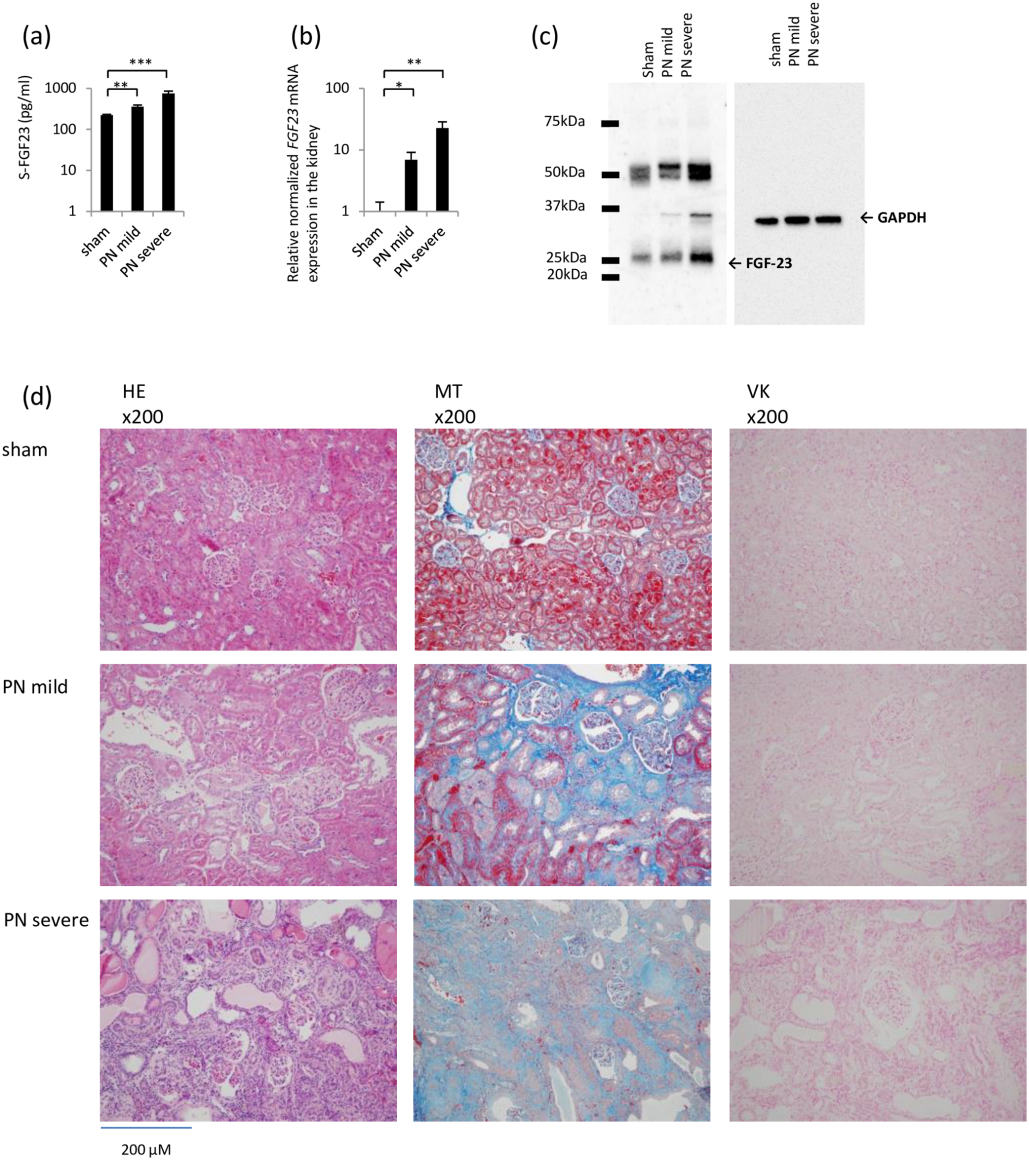
FGF23 expression in the partial nephrectomy rat model. (a) Serum FGF23 concentration. (b) FGF23 mRNA expression in the kidney. Sham group was used as a normalization control. (c) Western blot of FGF23 in the kidney. (d) Histology in the kidney. HE: hematoxylin-eosin staining. MT: Masson’s trichrome staining to evaluate fibrosis. VK: Von Kossa staining to evaluate calcification. *P<0.05, **P<0.01, ***P<0.001; sham group (n = 6), partial nephrectomy mild group (PN mild) (n = 6), partial nephrectomy severe group (PN severe) (n = 6).

The serum FGF23 levels detected in the PN mild and severe groups were higher than those detected in the sham group (Fig 8a). Although the expression of *FGF23* mRNA was barely detectable in the kidneys of the sham group, it was detected in both the PN mild and severe groups, and the levels were significantly higher than those in the sham group (Fig 8b). The expression of *FGF23* mRNA was not detectable in the livers of each group by real-time PCR (S3b Fig). The levels of FGF23 protein in the kidney were measured by Western blotting. The levels of FGF23 protein were elevated in the kidneys of the PN mild and severe groups (Fig 8c).

#### Renal histology

**Fig 9:**
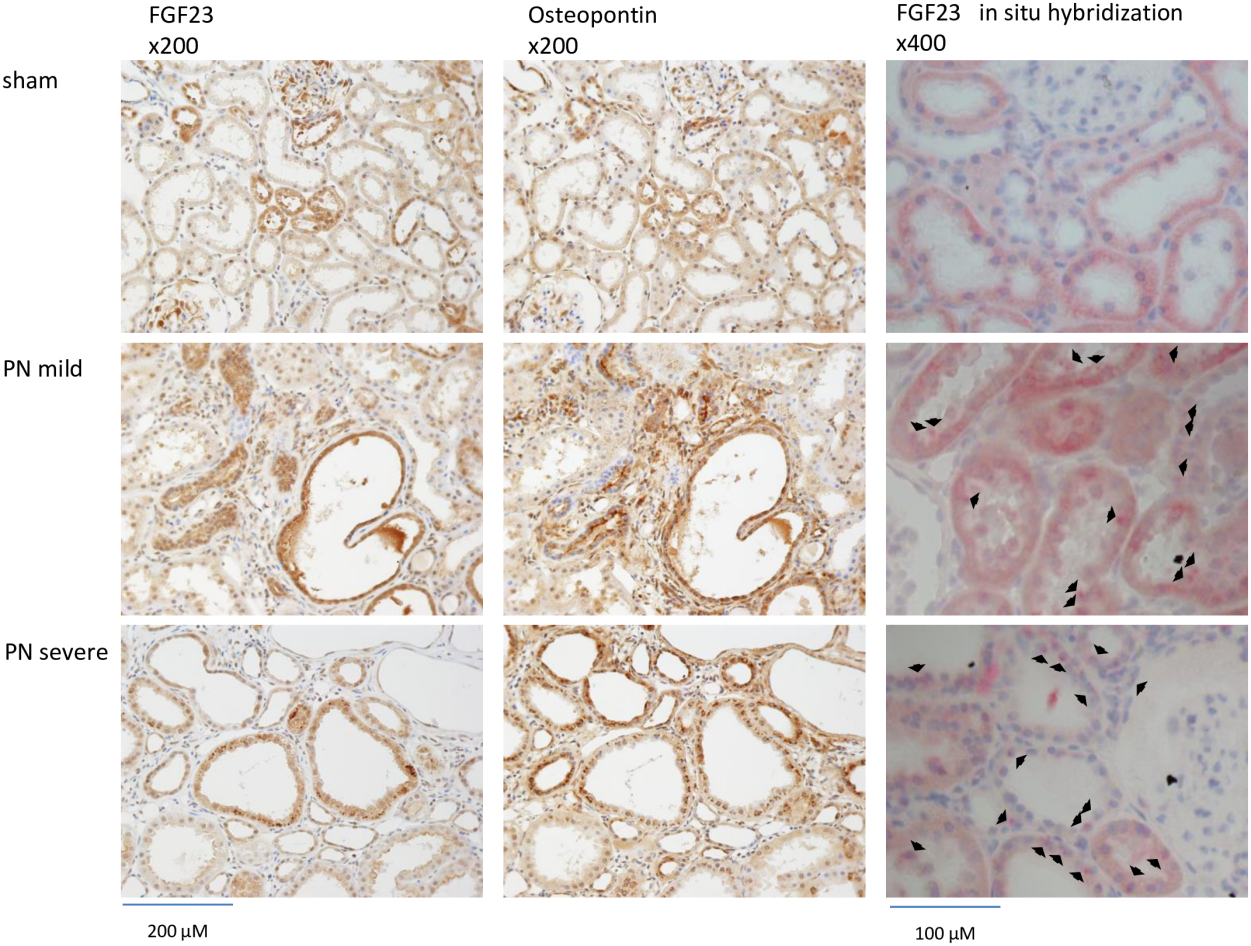
Immunohistochemistry and in situ hybridization in the kidney of partial nephrectomy rat model. FGF23: FGF23 immunohistochemistry (brown). Osteopontin: osteopontin immunohistochemistry (brown). ×200: high magnification. *FGF23* in situ hybridization (red spots; arrow: positive cells). ×400: high magnification.

In the PN mild and severe groups, we observed interstitial fibrosis, expansion of the renal tubule lumen and a decreased number of tubule cells (Fig 8d, HE and MT). Calcification was not observed by VK staining (Fig 8d, VK). Through immunohistochemical staining, FGF23 and osteopontin were detected in the expanded tubular cells (Fig 9, FGF23 and osteopontin). According to the *FGF23* in situ hybridization, *FGF23* mRNA was detected in the expanded tubular cells in the PN severe and mild groups (Fig 9, *FGF23* in situ hybridization).

### Rat model of DXR-induced renal failure

We conducted experiments in a rat model of DXR-induced renal failure as a third model of CKD to investigate whether FGF23 expression is a common phenomenon in renal tubulointerstitial disorder.

**Fig 10:**
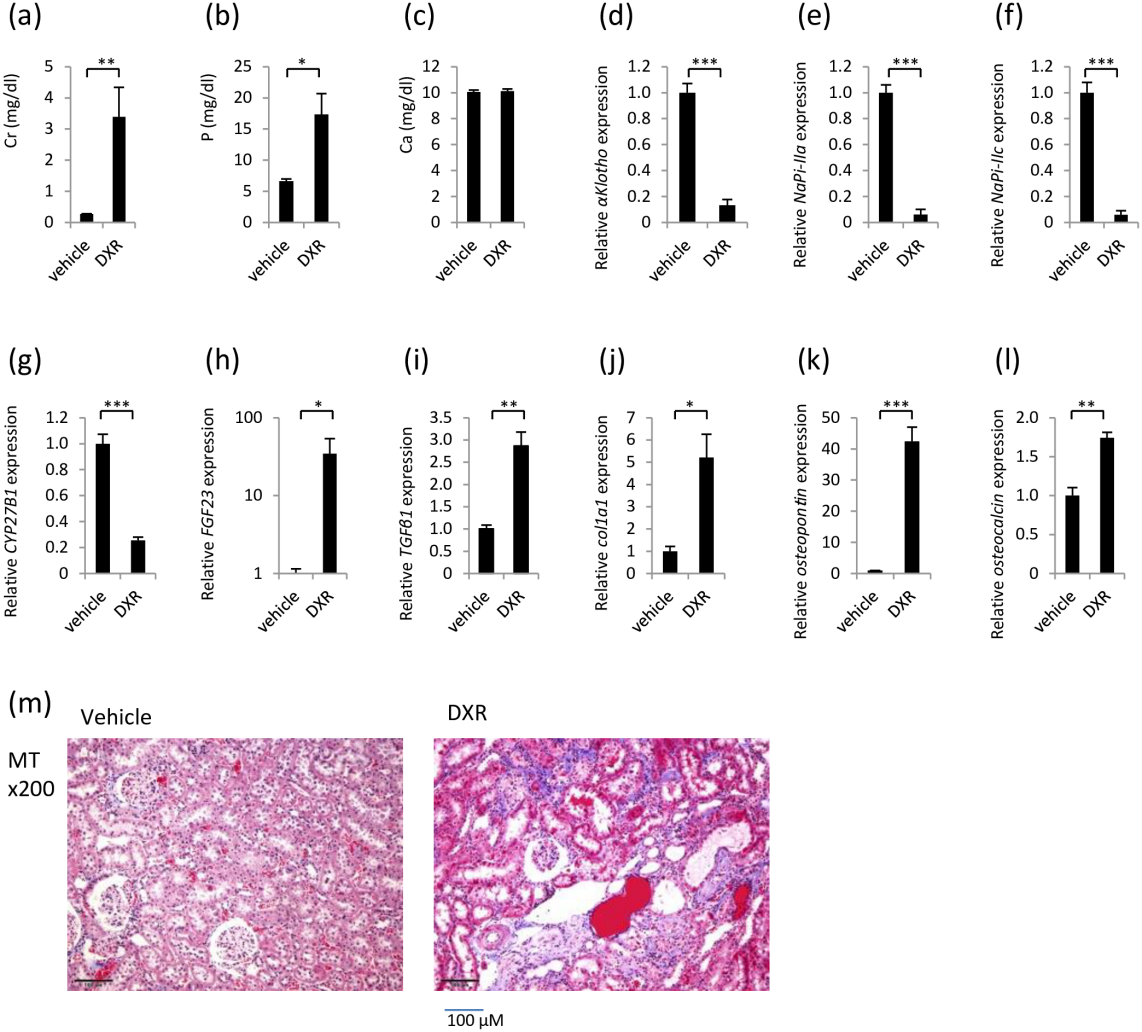
Rat model of DXR-induced renal failure. (a) Serum Cr concentration. (b) Serum P concentration. (c) Serum Ca concentration. (d) *αKlotho* mRNA expression in the kidney. (e) *NaPi-IIa* mRNA expression in the kidney. (f) *NaPi-IIc* mRNA expression in the kidney. (g) *CYP27B1* mRNA expression in the kidney. (h) *FGF23* mRNA expression in the kidney. (j) *TGFβ1* mRNA expression in the kidney. (j) *Col1a1* mRNA expression in the kidney. (k) *Osteopontin* mRNA expression in the kidney. (l) *Osteocalcin* mRNA expression in the kidney. (m) Histology in the kidney. MT: Masson’s trichrome staining to evaluate fibrosis. Each value represents the mean ± SEM. *P<0.05, **P<0.01, ***P<0.001,; vehicle group (n = 5) and DXR group (n = 5). (d-l) Vehicle group was used as a normalization control.

Regarding renal function, the serum Cr level was significantly higher in the DXR group than that in the vehicle group. The serum P level was significantly higher in the DXR group than that in the vehicle group. The serum Ca levels did not differ between the DXR group and vehicle group (Fig 10a–10c; these figures were reproduced from our previous report [22]). The *αKlotho*, *NaPi-IIa* and *NaPi-IIc* mRNAs were expressed as significantly lower levels in the kidneys of the DXR group than in the kidneys of the vehicle group (Fig 10d–10f). The expression of *CYP27B1* mRNA was significantly decreased in the DXR group (Fig 10g). In the DXR group, we observed interstitial fibrosis in the tubule lumen, an expansion of the renal tubule lumen and a decreased number of tubule cells (Fig 10i, MT).

The *FGF23* mRNA expression was measured in the kidney and was significantly higher in the DXR group than that in the vehicle group (Fig 10h). The *TGFβ1*, *Col1a1*, *osteopontin* and *osteocalcin* mRNAs were expressed at higher levels in the kidneys of the DXR group than in the kidneys of the vehicle group (Fig 10j–10l).

## Discussion

This study showed that FGF23 is produced in the kidneys of CKD model animals. The main site of FGF23 production has been reported to be the bone [3], and osteoblasts secrete FGF23 [6]. In addition to bone, FGF23 is also produced in the brain [28] in healthy subjects. Under pathological conditions, the FGF23 production sites include the kidney in rodent models of polycystic kidney disease (PKD) [29] and a Zucker diabetic fatty (ZDF) rat model of human type 2 diabetic nephropathy [30] and 5/6 nephrectomy [26] and the liver in diethyl-nitrosamine (DEN)-treated mice [31], patients on the Liver-Transplant Waiting List [31] at the time of liver failure, and vascular smooth muscle cells cultured in calcifying medium [24]. FGF23 protein has been detected in cells lining renal cysts in rodent models of PKD [29]. FGF23 expression has been speculated to be unrelated to serum and urinary P. Moreover, FGF23 produced in the kidneys in a PKD model and subjects with liver failure originates from the increased FGF23 concentration in the blood [31].

Vascular smooth muscle cells have been reported to transform and differentiate into osteoblast-like cells in response to high-P medium or CKD [10,24,32]. In this study, we assumed that vascular smooth muscle cells in the kidney secrete and express FGF23. However, according to the double staining of FGF23 and αSMA, αSMA-positive vascular smooth muscle cells do not express FGF23. The positive FGF23 and αSMA staining did not merge in these tissues. αSMA is an EMT marker. The *osteopontin*, *osteocalcin*, *Col1a1* (bone matrix markers) and *TGFβ1* (a fibrosis marker) mRNAs were expressed at higher levels in the kidneys from the CKD rats than those in the control kidneys. Thus, the renal tubular epithelial cells transformed into stromal-like cells that express FGF23. In particular, strong FGF23 staining was detected in the extended tubules in the kidneys of HP diet-fed rats, which showed positive Von Kossa staining. Since the kidney is a target organ of FGF23, positive signals were detected in the kidney through immunostaining. However, the *FGF23* mRNA expression was increased, and a positive signal of the *FGF23* mRNA was detected in the renal tubular epithelial cells that had attenuated their original function, suggesting that FGF23 is produced in these cells. In addition, focal FGF23 staining was previously observed in both proximal and distal tubular sections at a more advanced phase of the disease in a Zucker diabetic fatty (ZDF) rat diabetic nephropathy model [30]. The expression of *FGF23* mRNA was found of be increased in the following three rat models of CKD used in this study: HP diet-induced renal failure, partial nephrectomy, and DXR-induced renal failure. Based on these findings, renal expression of *FGF23* mRNA is a common phenomenon in CKD.

In the present study, Western blotting was performed and detected the levels of FGF23 protein, and an immunohistochemical study showed that FGF23 was located in the glomeruli and tubular cells in the NP sham group. In contrast, the *FGF23* mRNA expression was barely detectable by real-time PCR and in situ hybridization in the same group. Therefore, FGF23 proteins located in the glomerular capillary and tubular cells might originate from bone in subjects with normal kidney function. Since the kidney is a target organ of FGF23, FGF23 from bone might exist and bind the FGFR with αKlotho in the normal kidney.

The FGF23-αKlotho endocrine system is highly involved in bone mineral metabolism. Typically, FGF23 produced in bone mainly binds the membrane-type αKlotho and FGFR expressed in the kidney and parathyroid [5]. The function of the FGF23-αKlotho axis is to suppress NaPi-IIa and NaPi-IIc expression [6]. NaPi-IIa and NaPi-IIc are expressed on the luminal side of the proximal tubule and play major roles in P reabsorption. FGF23-αKlotho signaling inhibits NaPi-IIa and NaPi-IIc, promotes P excretion in the urine, and reduces the P level in the blood [3,33]. Furthermore, in the proximal tubules, the reduction in 1α-hydroxylase by FGF23-αKlotho signaling reduces 1,25(OH)_2_D_3_ synthesis and increases 24α-hydrolase expression, subsequently enhancing 25(OH)_2_D_3_ degradation. Consequently, the 1,25(OH)_2_D_3_ levels decrease [6]. Notably, 1,25(OH)_2_D_3_ increases FGF23 expression, suggesting that a negative feedback mechanism exists between 1,25(OH)_2_D_3_ and FGF23 [34,35].

We cannot easily explain the induction of FGF23 expression in the kidneys of the rats with CKD by the high serum P and 1,25(OH)_2_D_3_ levels in this study because the serum P and 1,25(OH)_2_D_3_ levels in the hemi-nephrectomized rats fed the HP diet were high, but normal and low levels were observed in the partial nephrectomy rat model, and HK2 cells stimulated with high-P or 1,25(OH)_2_D_3_ did not express *FGF23* mRNA. In all three animal CKD models, the expression of *TGFβ1* mRNA and FGF23 was elevated in the kidney. The *FGF23* mRNA was expressed in HK2 cells incubated with TGFβ1 in vitro. These results indicate that renal tubular epithelial cells are highly likely to express FGF23 following stimulation with TGFβ1.

In CKD, the blood FGF23 levels increase beginning at the early stage. FGF23 expression is increased by P loading, increased 1,25(OH)_2_D_3_ levels, decreased αKlotho expression, and reduced PTH levels [17–20]. In addition, αKlotho expression decreases during the early stage of CKD. The mechanism includes not only direct injury to the renal tubule cells that express αKlotho but also circulatory disorders, diabetes, uremic toxins, and oxidative stress [6,8,36]. A reduction in αKlotho expression induces FGF23 resistance, which increases the blood FGF23 level. The administration of an FGF23 neutralizing antibody in a rat model of early stage CKD increases the blood P concentration, reduces the fractional P excretion, and increases the 1,25(OH)_2_D_3_ levels [37]. Based on these findings, the elevation in the FGF23 levels during the early stage of CKD prevents the elevation of blood P levels. One possible mechanism is that the elevated FGF23 levels bind to reduced levels of αKlotho-FGFR, which reduces NaPi-IIa and NaPi-IIc expression. Consequently, urinary P excretion is promoted by suppressing P resorption in the tubules [3,33], thus attenuating the elevated blood P concentration in subjects with CKD.

In the present study, the renal expression of *αKlotho*, *NaPi-IIa* and *NaPi-IIc* mRNAs was reduced, the serum FGF23 and PTH levels were elevated, and FGF23 expression was increased in the calvaria and kidneys of the rat models of mild and severe CKD. The 24-hr U-P was higher in the HP groups than that in the NP groups and tended to be higher in the PN mild and severe groups than that in the sham group. FGF23 expressed in the kidney might play a role in the control of blood and urine P levels and acts on αKlotho and FGFR as a paracrine and autocrine factor to directly affect P resorption in the kidneys of CKD rats.

In the partial nephrectomy rat model, the mRNA expression of *CYP27B1*, which encodes 1α-hydroxylase, was increased, whereas the serum 1,25(OH)_2_D_3_ concentration was decreased. Thus, we detected a dissociation between *CYP27B1* expression and the 1,25(OH)_2_D_3_ levels. This dissociation might have been caused by the reduced kidney volume in the PN groups. Unexpectedly, during the early stage of HP diet-induced CKD, the 1,25(OH)_2_D_3_ concentration was increased, and the expression of the *CYP27B1* mRNA was elevated in the partial nephrectomy rats fed the HP diet. Since this finding cannot be explained by FGF23 alone, a complex interaction likely exists among Ca, P and PTH in the blood and other molecules involved in mineral metabolism.

## Conclusions

In CKD rat models, *FGF23* mRNA is expressed in the kidney, and the FGF23 protein is expressed at high levels in osteopontin-positive renal tubule epithelium cells likely via TGFβ1 stimulation. FGF23 produced in the kidney might contribute to P metabolism in subjects with CKD.

## Supporting information

S1 Fig *FGF23* mRNA expression in bone. The *FGF23* mRNA level in the femur correlates with the level in the calvaria (R = 0.87, n = 9).
(TIF)

S2 Fig Negative and positive controls. **(a) Negative and positive controls for the in situ hybridization.** Red spots are positive for the indicated mRNA. Red patchy areas are negative for the indicated mRNA. **(b) Negative and positive controls for the FGF23 immunostaining**. FGF23 staining is not observed in the liver. FGF23 staining is observed in osteocytes (brown spots; arrow: positive cells). **(c) FGF23 immunostaining in the kidney using only the secondary antibody.** No positive staining was observed in the kidney in the absence of primary antibodies against FGF23 and osteopontin. ×200, ×400: high magnification.
(TIF)

S3 Fig *FGF23* mRNA expression in the liver. **(a) Hemi-Nephrectomized Rats Fed a High-P diet.** NP sham group (n = 4), NP Nx group (n = 4), HP sham group (n = 4) and HP Nx group (n = 4). **(b) Partial Nephrectomy Rat Model**. Wild type group (n = 2), Sham group (n = 4), PN mild group (n = 4), PN severe group (n = 4). Bone group was used as a normalization control. Each value represents the mean ± SEM. The expression of *FGF23* mRNA was not detectable in the livers of each group by real-time PCR.
(TIF)

## References

[1] Consortium A. Autosomal dominant hypophosphataemic rickets is associated with mutations in FGF23. Nat Genet. 2000;26: 345–348. doi: 10.1038/81664 11062477 10.1038/81664

[2] Shimada T, Mizutani S, Muto T, Yoneya T, Hino R, Takeda S, et al Cloning and characterization of FGF23 as a causative factor of tumor-induced osteomalacia. Proc Natl Acad Sci USA. 2001;98: 6500–6505. doi: 10.1073/pnas.101545198 11344269 10.1073/pnas.101545198 PMC33497

[3] Larsson T, Marsell R, Schipani E, Ohlsson C, Ljunggren O, Tenenhouse HS, et al Transgenic mice expressing fibroblast growth factor 23 under the control of the alpha1(I) collagen promoter exhibit growth retardation, osteomalacia, and disturbed phosphate homeostasis. Endocrinology. 2004;145: 3087–3094. doi: 10.1210/en.2003-1768 14988389 10.1210/en.2003-1768

[4] Shimada T, Kakitani M, Yamazaki Y, Hasegawa H, Takeuchi Y, Fujita T, et al Targeted ablation of Fgf23 demonstrates an essential physiological role of FGF23 in phosphate and vitamin D metabolism. J Clin Invest. 2004;113: 561–568. doi: 10.1172/JCI19081 14966565 10.1172/JCI19081 PMC338262

[5] Kurosu H, Ogawa Y, Miyoshi M, Yamamoto M, Nandi A, Rosenblatt KP, et al Regulation of fibroblast growth factor-23 signaling by klotho. J Biol Chem. 2006;281: 6120–6123. doi: 10.1074/jbc.C500457200 16436388 10.1074/jbc.C500457200 PMC2637204

[6] Hu MC, Shiizaki K, Kuro-o M, Moe OW. Fibroblast growth factor 23 and Klotho: physiology and pathophysiology of an endocrine network of mineral metabolism. Annu Rev Physiol. 2013;75: 503–533. doi: 10.1146/annurev-physiol-030212-183727 23398153 10.1146/annurev-physiol-030212-183727 PMC3770142

[7] Sugiura H, Yoshida T, Tsuchiya K, Mitobe M, Nishimura S, Shirota S, et al Klotho reduces apoptosis in experimental ischaemic acute renal failure. Nephrol Dial Transplant. 2005;20: 2636–2645. doi: 10.1093/ndt/gfi165 16204278 10.1093/ndt/gfi165

[8] Sugiura H, Yoshida T, Mitobe M, Yoshida S, Shiohira S, Nitta K, et al Klotho reduces apoptosis in experimental ischaemic acute kidney injury via HSP-70. Nephrol Dial Transplant. 2010;25: 60–68. doi: 10.1093/ndt/gfp451 19745103 10.1093/ndt/gfp451

[9] Sugiura H, Yoshida T, Shiohira S, Kohei J, Mitobe M, Kurosu H, et al Reduced Klotho expression level in kidney aggravates renal interstitial fibrosis. Am J Physiol Renal Physiol. 2012;302: F1252–1264. doi: 10.1152/ajprenal.00294.2011 22338084 10.1152/ajprenal.00294.2011

[10] Hu MC, Shi M, Zhang J, Quinones H, Griffith C, Kuro-o M, et al Klotho deficiency causes vascular calcification in chronic kidney disease. J Am Soc Nephrol. 2011;22: 124–136. doi: 10.1681/ASN.2009121311 21115613 10.1681/ASN.2009121311 PMC3014041

[11] Sakan H, Nakatani K, Asai O, Imura A, Tanaka T, Yoshimoto S, et al Reduced renal alpha-Klotho expression in CKD patients and its effect on renal phosphate handling and vitamin D metabolism. PLoS One. 2014;9: e86301 doi: 10.1371/journal.pone.0086301 24466013 10.1371/journal.pone.0086301 PMC3900516

[12] Shimamura Y, Hamada K, Inoue K, Ogata K, Ishihara M, Kagawa T, et al Serum levels of soluble secreted alpha-Klotho are decreased in the early stages of chronic kidney disease, making it a probable novel biomarker for early diagnosis. Clin Exp Nephrol. 2012;16: 722–729. doi: 10.1007/s10157-012-0621-7 22457086 10.1007/s10157-012-0621-7

[13] Isakova T, Wahl P, Vargas GS, Gutierrez OM, Scialla J, Xie H, et al Fibroblast growth factor 23 is elevated before parathyroid hormone and phosphate in chronic kidney disease. Kidney Int. 2011;79: 1370–1378. doi: 10.1038/ki.2011.47 21389978 10.1038/ki.2011.47 PMC3134393

[14] Gutierrez OM, Mannstadt M, Isakova T, Rauh-Hain JA, Tamez H, Shah A, et al Fibroblast growth factor 23 and mortality among patients undergoing hemodialysis. N Engl J Med. 2008;359: 584–592. doi: 10.1056/NEJMoa0706130 18687639 10.1056/NEJMoa0706130 PMC2890264

[15] Faul C, Amaral AP, Oskouei B, Hu MC, Sloan A, Isakova T, et al FGF23 induces left ventricular hypertrophy. J Clin Invest. 2011;121: 4393–4408. doi: 10.1172/JCI46122 21985788 10.1172/JCI46122 PMC3204831

[16] Di Marco GS, Reuter S, Kentrup D, Grabner A, Amaral AP, Fobker M, et al Treatment of established left ventricular hypertrophy with fibroblast growth factor receptor blockade in an animal model of CKD. Nephrol Dial Transplant. 2014;29: 2028–2035. doi: 10.1093/ndt/gfu190 24875663 10.1093/ndt/gfu190 PMC4425841

[17] Martin A, David V, Quarles LD. Regulation and function of the FGF23/klotho endocrine pathways. Physiol Rev. 2012;92: 131–155. doi: 10.1152/physrev.00002.2011 22298654 10.1152/physrev.00002.2011 PMC3306265

[18] Lopez I, Rodriguez-Ortiz ME, Almaden Y, Guerrero F, de Oca AM, Pineda C, et al Direct and indirect effects of parathyroid hormone on circulating levels of fibroblast growth factor 23 in vivo. Kidney Int. 2011;80: 475–482. doi: 10.1038/ki.2011.107 21525854 10.1038/ki.2011.107

[19] Rodriguez-Ortiz ME, Lopez I, Munoz-Castaneda JR, Martinez-Moreno JM, Ramirez AP, Pineda C, et al Calcium deficiency reduces circulating levels of FGF23. J Am Soc Nephrol. 2012;23: 1190–1197. doi: 10.1681/ASN.2011101006 22581996 10.1681/ASN.2011101006 PMC3380648

[20] Haussler MR, Whitfield GK, Kaneko I, Forster R, Saini R, Hsieh JC, et al The role of vitamin D in the FGF23, klotho, and phosphate bone-kidney endocrine axis. Rev Endocr Metab Disord. 2012;13: 57–69. doi: 10.1007/s11154-011-9199-8 21932165 10.1007/s11154-011-9199-8 PMC3288475

[21] Nagano N, Miyata S, Abe M, Kobayashi N, Wakita S, Yamashita T, et al Effect of manipulating serum phosphorus with phosphate binder on circulating PTH and FGF23 in renal failure rats. Kidney Int. 2006;69: 531–537. doi: 10.1038/sj.ki.5000020 16395276 10.1038/sj.ki.5000020

[22] Sugiura H, Yoshida T, Mitobe M, Shiohira S, Nitta K, Tsuchiya K. Recombinant human erythropoietin mitigates reductions in renal klotho expression. Am J Nephrol. 2010;32: 137–144. doi: 10.1159/000315864 20606417 10.1159/000315864

[23] Noiri E, Nagano N, Negishi K, Doi K, Miyata S, Abe M, et al Efficacy of darbepoetin in doxorubicin-induced cardiorenal injury in rats. Nephron Exp Nephrol. 2006;104: e6–e14. doi: 10.1159/000093258 16707910 10.1159/000093258

[24] Zhu D, Mackenzie NC, Millan JL, Farquharson C, MacRae VE. A protective role for FGF-23 in local defence against disrupted arterial wall integrity? Mol Cell Endocrinol. 2013;372: 1–11. doi: 10.1016/j.mce.2013.03.008 23523568 10.1016/j.mce.2013.03.008 PMC3725806

[25] Lee JW, Yamaguchi A, Iimura T. Functional heterogeneity of osteocytes in FGF23 production: the possible involvement of DMP1 as a direct negative regulator. Bonekey Rep. 2014;3: 543 doi: 10.1038/bonekey.2014.38 24991406 10.1038/bonekey.2014.38 PMC4078414

[26] Mace ML, Gravesen E, Nordholm A, Hofman-Bang J, Secher T, Olgaard K, et al Kidney fibroblast growth factor 23 does not contribute to elevation of its circulating levels in uremia. Kidney Int. 2017;92: 165–178. doi: 10.1016/j.kint.2017.01.015 28341272 10.1016/j.kint.2017.01.015

[27] Usui N, Watanabe K, Ono K, Tomita K, Tamamaki N, Ikenaka K, et al Role of motoneuron-derived neurotrophin 3 in survival and axonal projection of sensory neurons during neural circuit formation. Development. 2012;139: 1125–1132. doi: 10.1242/dev.069997 22318233 10.1242/dev.069997

[28] Yamashita T, Yoshioka M, Itoh N. Identification of a novel fibroblast growth factor, FGF-23, preferentially expressed in the ventrolateral thalamic nucleus of the brain. Biochem Biophys Res Commun. 2000;277: 494–498. doi: 10.1006/bbrc.2000.3696 11032749 10.1006/bbrc.2000.3696

[29] Spichtig D, Zhang H, Mohebbi N, Pavik I, Petzold K, Stange G, et al Renal expression of FGF23 and peripheral resistance to elevated FGF23 in rodent models of polycystic kidney disease. Kidney Int. 2014;85: 1340–1350. doi: 10.1038/ki.2013.526 24402093 10.1038/ki.2013.526

[30] Zanchi C, Locatelli M, Benigni A, Corna D, Tomasoni S, Rottoli D, et al Renal expression of FGF23 in progressive renal disease of diabetes and the effect of ACE inhibitor. PLoS One. 2013;8: e70775 doi: 10.1371/journal.pone.0070775 23967103 10.1371/journal.pone.0070775 PMC3743899

[31] Prie D, Forand A, Francoz C, Elie C, Cohen I, Courbebaisse M, et al Plasma fibroblast growth factor 23 concentration is increased and predicts mortality in patients on the liver-transplant waiting list. PLoS One. 2013;8: e66182 doi: 10.1371/journal.pone.0066182 23825530 10.1371/journal.pone.0066182 PMC3692511

[32] Shimokado A, Sun Y, Nakanishi M, Sato F, Oikawa K, Akasaka T, et al Smad3 plays an inhibitory role in phosphate-induced vascular smooth muscle cell calcification. Exp Mol Pathol. 2014;97: 458–464. doi: 10.1016/j.yexmp.2014.10.005 25303897 10.1016/j.yexmp.2014.10.005

[33] Olauson H, Lindberg K, Amin R, Jia T, Wernerson A, Andersson G, et al Targeted deletion of Klotho in kidney distal tubule disrupts mineral metabolism. J Am Soc Nephrol. 2012;23: 1641–1651. doi: 10.1681/ASN.2012010048 22878961 10.1681/ASN.2012010048 PMC3458458

[34] Forster RE, Jurutka PW, Hsieh JC, Haussler CA, Lowmiller CL, Kaneko I, et al Vitamin D receptor controls expression of the anti-aging klotho gene in mouse and human renal cells. Biochem Biophys Res Commun. 2011;414: 557–562. doi: 10.1016/j.bbrc.2011.09.117 21982773 10.1016/j.bbrc.2011.09.117 PMC3209523

[35] Saji F, Shigematsu T, Sakaguchi T, Ohya M, Orita H, Maeda Y, et al Fibroblast growth factor 23 production in bone is directly regulated by 1{alpha},25-dihydroxyvitamin D, but not PTH. Am J Physiol Renal Physiol. 2010;299: F1212–1217. doi: 10.1152/ajprenal.00169.2010 20739393 10.1152/ajprenal.00169.2010

[36] Mitobe M, Yoshida T, Sugiura H, Shirota S, Tsuchiya K, Nihei H. Oxidative stress decreases klotho expression in a mouse kidney cell line. Nephron Exp Nephrol. 2005;101: e67–74. doi: 10.1159/000086500 15976510 10.1159/000086500

[37] Hasegawa H, Nagano N, Urakawa I, Yamazaki Y, Iijima K, Fujita T, et al Direct evidence for a causative role of FGF23 in the abnormal renal phosphate handling and vitamin D metabolism in rats with early-stage chronic kidney disease. Kidney Int. 2010;78: 975–980. doi: 10.1038/ki.2010.313 20844473 10.1038/ki.2010.313

