# Supplementary figures and images for: Fibroblast growth factor 23 is upregulated in the kidney in a chronic kidney disease rat model
Source: PLoS One. 2018 Mar 8;13(3):e0191706. doi: 10.1371/journal.pone.0191706 (PMC5843171; doi:10.1371/journal.pone.0191706)

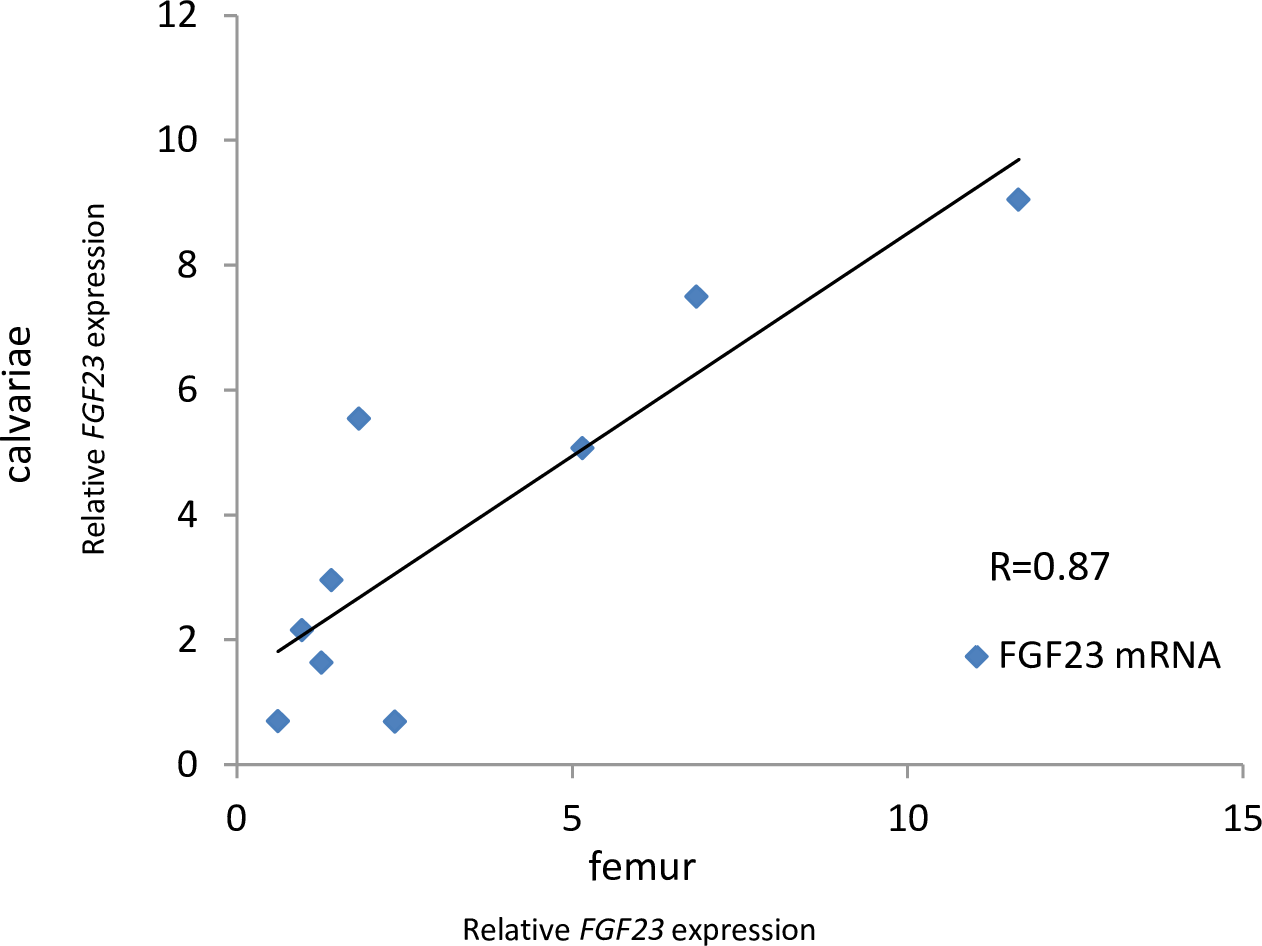

Supplement: S1 Fig — The FGF23 mRNA level in the femur correlates with the level in the calvaria (R = 0.87, n = 9). (TIF) [file pone.0191706.s001.tif]

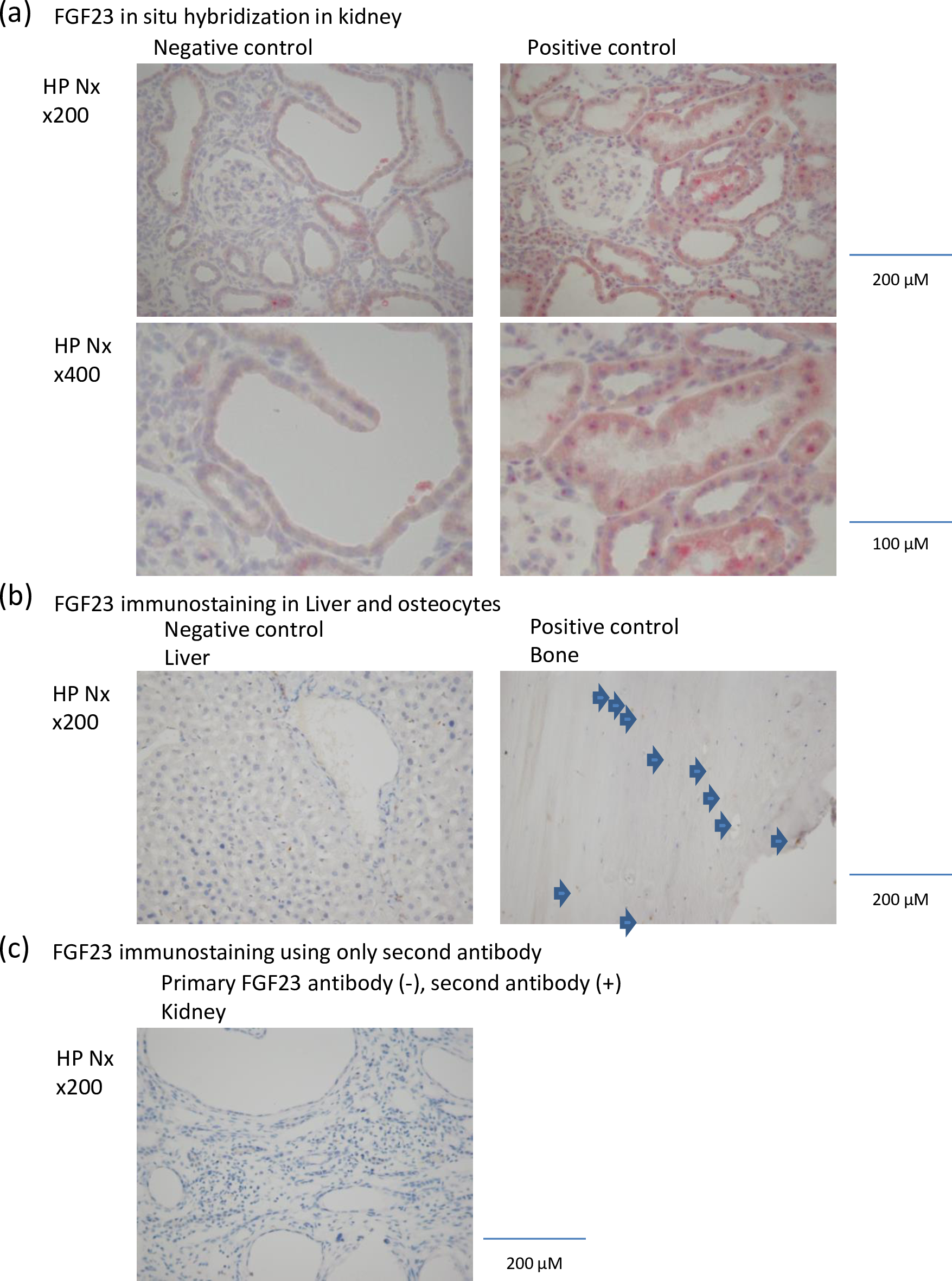

Supplement: S2 Fig — (a) Negative and positive controls for the in situ hybridization. Red spots are positive for the indicated mRNA. Red patchy areas are negative for the indicated mRNA. (b) Negative and positive controls for the FGF23 immunostaining. FGF23 staining is not observed in the liver. FGF23 staining is observed in osteocytes (brown spots; arrow: positive cells). (c) FGF23 immunostaining in the kidney using only the secondary antibody. No positive staining was observed in the kidney in the absence of primary antibodies against FGF23 and osteopontin. ×200, ×400: high magnification. (TIF) [file pone.0191706.s002.tif]
